# Dietary Modulation of Inflammatory and Oxidative Pathways in Type 2 Diabetes: Biomarkers and Cardiorenal Outcomes

**DOI:** 10.3390/nu18101592

**Published:** 2026-05-16

**Authors:** Carlo Domenico Maida, Stefania Scaglione, Rosario Luca Norrito, Mario Daidone, Gaetano Pacinella, Antonino Marchese, Filippo Vutano, Giuliano Cassataro, Luigi Dell’Ajra, Sergio Ferrantelli, Alessandro Del Cuore, Chiara Maurici, Gabriele Vassallo, Antonino Tuttolomondo

**Affiliations:** 1 Department of Internal Medicine, S. Elia Hospital, 93100 Caltanissetta, Italy; 2 Molecular and Clinical Medicine PhD Program, University of Palermo, 90127 Palermo, Italypacinella66@gmail.com (G.P.);; 3 Internal Medicine and Stroke Care Ward, Department of Health Promotion, Mother and Child Care, Internal Medicine and Medical Specialities, University of Palermo, 90127 Palermo, Italy; 4 Department of Internal Medicine, Buccheri La Ferla Hospital, 90100 Palermo, Italy; 5 Medicine Unit, Fondazione G. Giglio, 90015 Cefalù, Italy; 6 Cardiology Unit, Cervello Hospital, A.O. Ospedali Riuniti Villa Sofia-Cervello, 90146 Palermo, Italy; 7 Geriatrics Unit, Garibaldi-Nesima Hospital, ARNAS Garibaldi, 95122 Catania, Italy; chiaramaurici.92@gmail.com; 8 Department of Internal Medicine, Barone Lombardo Hospital, 92024 Canicattì, Italy

**Keywords:** type 2 diabetes mellitus, inflammation, oxidative stress, nutrition, Mediterranean diet, biomarkers, diabetic kidney disease, cardiovascular risk

## Abstract

Type 2 diabetes mellitus is a relevant cardio–renal–metabolic disorder in which chronic low-grade inflammation and oxidative stress have a crucial function in linking insulin resistance, endothelial dysfunction, β-cell impairment, and progressive organ injury. In this context, nutrition has emerged as a key modifiable determinant of metabolic homeostasis, capable of influencing inflammatory signalling, redox balance, mitochondrial function, and gut microbiota–host interactions. The objective of this review is to critically summarise the mechanistic connections among inflammation, oxidative stress, and diabetes progression, and to investigate how dietary factors and patterns, as well as nutrition-responsive biomarkers, influence these pathways and their cardiorenal consequences. We discuss the effects of macronutrient quality, dietary fibre, fatty acids, polyphenols, and specific micronutrients, including vitamin C, vitamin E, selenium, zinc, and magnesium, as well as the role of Mediterranean, DASH, and plant-based diets in improving glycaemic control, endothelial function, and cardio-renal risk profiles. We also summarise established and emerging biomarkers of inflammation and oxidative stress that may improve risk stratification and the evaluation of nutrition-based interventions. Overall, current evidence supports a shift from a purely glucose-centred approach toward an integrated model in which dietary modulation of inflammatory and oxidative pathways helps reduce cardiovascular and renal risk. However, heterogeneity of interventions, variability in biomarker assessment, and interindividual differences in dietary response represent major limitations. Future research should focus on biomarker-informed, precision-oriented nutritional approaches integrated within contemporary cardio–renal–metabolic care.

## 1. Introduction

### 1.1. Diabetes and Long-Term Cardiovascular and Renal Burden

Diabetes mellitus (DM) is one of the most prevalent metabolic disorders worldwide, with type 2 diabetes mellitus (T2DM) representing the dominant clinical form. According to the World Health Organisation, its global incidence continues to rise, driven by increasing rates of obesity, sedentary lifestyles, and population ageing [1]. Beyond its metabolic nature, DM should be considered a systemic disease characterised by progressive multisystem involvement and long-term complications.

Although blood glucose levels define the diagnosis, systemic complications—particularly cardiovascular and renal—are the major determinants of morbidity and mortality. Patients with DM have a markedly increased risk of developing atherosclerotic cardiovascular disease, heart failure, and chronic kidney disease (CKD) [2]. Notably, these complications often remain clinically silent until advanced stages.

Diabetic kidney disease represents the leading cause of end-stage CKD, while cardiovascular events account for the majority of deaths in diabetic populations [3]. The frequent coexistence of cardiovascular and renal dysfunction suggests shared pathogenic pathways, including hemodynamic stress, oxidative injury, and chronic low-grade inflammation.

Despite significant advances in glucose-lowering and cardiorenal-protective therapies, patients with DM continue to exhibit a higher residual cardiovascular risk compared with non-diabetic individuals, indicating that the underlying metabolic, oxidative, and inflammatory mechanisms are not yet fully understood.

### 1.2. Inflammation and Oxidative Stress as Convergent Mechanisms

Oxidative stress and chronic inflammation play a central role in the progression of diabetes and its long-term complications. Insulin resistance, persistent hyperglycaemia, and dyslipidaemia promote mitochondrial dysfunction, leading to excessive production of reactive oxygen species (ROS). This redox imbalance activates pro-inflammatory signalling pathways, including nuclear factor kappa B (NF-κB), which in turn amplifies cytokine production and endothelial activation [4,5].

These processes contribute to endothelial dysfunction, vascular remodelling, glomerular injury, and tissue fibrosis. In addition, oxidative stress reduces nitric oxide bioavailability and promotes lipid peroxidation, further impairing vascular homeostasis.

Chronic exposure to hyperglycaemia and lipotoxicity also favours the formation of advanced glycation end-products (AGEs) and activation of protein kinase C pathways, which further sustain inflammatory cascades and cellular damage. Circulating biomarkers such as high-sensitivity C-reactive protein (hs-CRP), interleukins, and tumour necrosis factor alpha (TNF-α) have been consistently associated with adverse cardiovascular and renal outcomes [4,5].

Taken together, these findings suggest that glycaemic control alone may be insufficient to halt disease progression, whereas strategies targeting inflammation and oxidative stress may provide additional prognostic benefit.

### 1.3. Rationale for Nutrition-Based Modulation

Growing evidence highlights the pivotal role of nutrition in modulating metabolic and inflammatory pathways. Dietary composition influences glycaemic control, insulin signalling, lipid metabolism, gut microbiota composition, and redox balance.

Specific dietary components—including polyphenols; *n*-3 polyunsaturated fatty acids (PUFAs), such as plant-derived alpha-linolenic acid (ALA) and marine-derived eicosapentaenoic acid (EPA) and docosahexaenoic acid (DHA); dietary fibre; micronutrients such as vitamin C, vitamin E, selenium, zinc, and magnesium; and plant-derived antioxidants—play an anti-inflammatory and antioxidant role by supporting endogenous antioxidant defences, controlling lipid peroxidation, influencing inflammatory signalling, and defending endothelial function. In contrast, dietary patterns rich in refined carbohydrates, saturated fats, and ultra-processed foods are associated with metabolic dysfunction and systemic inflammation [6].

Among dietary models, the Mediterranean and plant-based diets have shown consistent beneficial effects on glycaemic control, endothelial function, oxidative stress, and inflammatory biomarkers [7,8]. Furthermore, emerging data from metabolomics, nutrigenomics, and microbiome research suggest that individual responses to diet are highly heterogeneous, supporting the concept of precision nutrition.

However, despite these advances, the mechanistic pathways underlying nutrition-mediated metabolic modulation remain incompletely understood and warrant further investigation. Accordingly, this review aims to synthesise mechanistic and clinical evidence on how dietary components and patterns influence inflammatory and oxidative pathways in T2DM, with a focus on biomarker-based risk stratification and cardiovascular and renal outcomes.

## 2. Pathophysiological Mechanisms

### 2.1. Chronic Low-Grade Inflammation and Insulin Resistance

This subsection focuses on the immunometabolic genesis of insulin resistance in type 2 diabetes mellitus (T2DM). Its purpose is to define how adipose tissue dysfunction, innate immune activation, cytokine networks, adipokines, hepatic insulin resistance, and gut-derived inflammatory signals affect insulin signalling. Redox mechanisms are mentioned here only where they participate in this immune-metabolic process; their vascular and endothelial consequences are addressed separately in Section 2.2.

Insulin resistance represents a central pathogenic driver in T2DM and is strongly associated with chronic inflammation. Following nutrient intake, intestinal absorption of carbohydrates and subsequent hepatic metabolic processing increase plasma glucose levels, stimulating insulin secretion from pancreatic β-cells. In insulin-resistant states, this compensatory mechanism is accompanied by increased production of pro-inflammatory cytokines, including interleukin-6 (IL-6), tumour necrosis factor alpha (TNF-α), interleukin-1 (IL-1), and plasminogen activator inhibitor-1 (PAI-1).

Adipose tissue plays a pivotal role in this process by secreting adipokines and cytokines that sustain a chronic inflammatory state. Dysregulation of signalling pathways—including activation of NF-κB, upregulation of TNF-related pathways, and suppression of peroxisome proliferator-activated receptors—leads to impaired translocation of glucose transporter type 4 (GLUT-4). The resulting decrease in glucose uptake contributes to hyperinsulinaemia and perpetuates a metabolic–inflammatory feedback loop.

Early activation of innate immune responses further amplifies this process. Neutrophil infiltration into adipose tissue promotes insulin resistance through the release of elastase, myeloperoxidase, and pro-inflammatory cytokines. These mediators not only damage insulin-sensitive tissues but also favour macrophage polarisation toward the pro-inflammatory M1 phenotype, thereby sustaining chronic inflammation [9].

A critical pathogenic determinant lies in the imbalance between macrophage subtypes. M1 macrophages, primarily relying on glycolytic metabolism, promote inflammation through continuous cytokine production, whereas M2 macrophages exert anti-inflammatory and tissue-reparative functions. Disruption of this balance results in a self-amplifying cycle of inflammation, immune cell recruitment, and metabolic dysfunction, ultimately driving progression from insulin resistance to overt metabolic disease [9].

Lipid-derived factors influence metabolic signalling in a context-dependent manner. Arachidonic acid may serve as a precursor to eicosanoids and other bioactive lipid mediators with pro-inflammatory function. In contrast, oleic acid, particularly within monounsaturated fat-rich dietary patterns, is generally regarded as metabolically neutral or protective. Therefore, these fatty acids should not be considered as having equivalent effects on insulin signalling or metabolic dysfunction. When lipid-derived inflammatory signalling hampers insulin action, the key downstream defect mainly involves inhibitory serine phosphorylation and functional injury of insulin receptor substrate-1 (IRS-1), rather than direct phosphorylation of the insulin receptor itself.

The progressive increase in circulating insulin levels due to peripheral resistance leads to sustained stimulation of adipose and skeletal muscle tissue, resulting in enhanced secretion of adipokines and myokines that maintain a chronic low-grade inflammatory state. Leptin, although primarily involved in appetite regulation, assumes a pro-inflammatory role in leptin resistance, where its signalling capacity is impaired. At the same time, its ability to activate the NF-κB and JAK pathways is preserved.

Among emerging adipokines, asprosin has been proposed as a potential modulator of glucose metabolism. Emerging evidence from human studies is largely observational and suggests associations between circulating asprosin concentrations and obesity, insulin resistance, T2DM, and glycaemic indices; however, these findings remain limited, partly heterogeneous, and do not prove any causality. By contrast, mechanistic evidence derives largely from animal and cellular models, in which asprosin has been reported to promote hepatic glucose release through cyclic adenosine monophosphate (cAMP)–protein kinase A (PKA) signalling and to influence endoplasmic reticulum stress, fatty acid β-oxidation, lipogenesis, inflammation, and fibrogenesis. Accordingly, asprosin should be estimated as a candidate adipokine involved in metabolic dysregulation rather than a fully established pathogenic mediator in humans [10].

Another relevant adipokine is retinol-binding protein 4 (RBP4), which has been associated with insulin resistance and T2DM. RBP4 impairs insulin signalling in skeletal muscle and adipose tissue by activating the toll-like receptor 4 (TLR4) and c-Jun *N*-terminal kinase (JNK) pathways, thereby promoting inflammatory cytokine production and inhibiting GLUT-4 translocation.

Additional cytokines, including interleukins IL-17, IL-18, IL-33, IL-37, and IL-38, contribute to the inflammatory milieu. In particular, reduced IL-33 signalling has been associated with impaired M2 macrophage polarisation and increased TNF-α and IL-6 levels, further exacerbating insulin resistance.

Hepatic insulin resistance plays a central role in systemic metabolic dysfunction. Under physiological conditions, insulin suppresses hepatic glucose production by inhibiting gluconeogenesis and glycogenolysis while promoting glycogen synthesis. However, in insulin-resistant states, this regulatory balance is disrupted—a phenomenon known as “selective hepatic insulin resistance.” Impaired signalling along the phosphatidylinositol 3-kinase–protein kinase B (PI3K–Akt) pathway results in persistent activation of gluconeogenic transcription factors, such as forkhead box protein O1 (FOXO1), thereby sustaining hepatic glucose output despite hyperinsulinaemia [11].

Simultaneously, hepatic insulin resistance promotes lipid dysregulation. Increased free fatty acid flux from adipose tissue enhances intrahepatic triglyceride accumulation, contributing to steatosis. Lipid intermediates such as diacylglycerols and ceramides further affect insulin signalling by activating PKC and inflammatory kinase pathways, which stimulate inhibitory serine phosphorylation and functional impairment of IRS-1. This mechanism reduces downstream PI3K-Akt signalling, thereby establishing a vicious cycle of metabolic dysfunction. These processes are further exacerbated by mitochondrial dysfunction and oxidative stress.

From a systemic perspective, hepatic insulin resistance contributes to dyslipidaemia by increasing very-low-density lipoprotein (VLDL) secretion, thereby linking glucose and lipid metabolism within a unified pathophysiological framework [11].

The intestinal microbiota has also emerged as a key modulator of insulin sensitivity through a broader microbiota–host signalling network rather than through lipopolysaccharide (LPS)–toll-like receptor 4 (TLR4) activation alone. Dysbiosis, characterised by an imbalance in microbial composition and decreased barrier integrity, may stimulate the translocation of microbial components, such as LPS, thereby contributing to metabolic endotoxemia and TLR4-dependent inflammatory signalling. However, this axis should be considered as only one component of a more complex microbial-metabolic system [12,13,14].

Microbial metabolites also modulate glucose homeostasis and the inflammatory background. Short-chain fatty acids (SCFAs), including acetate, propionate, and butyrate, produced by the fermentation of dietary fibre, protect intestinal barrier integrity, modulate immune cell activation, and interact with host receptors involved in appetite regulation, incretin secretion, and insulin sensitivity. In parallel, gut microbiota-dependent bile acid transformation affects host metabolism through farnesoid X receptor (FXR) and Takeda G protein-coupled receptor 5 (TGR5) signalling, thereby influencing glucose and lipid metabolism, energy expenditure, and inflammation. Other microbial products, including indole derivatives and trimethylamine-related metabolites, may further contribute to epithelial integrity, systemic inflammation, endothelial function, and cardiometabolic risk. Therefore, in T2DM, dysbiosis should be regarded as a multidimensional contributor to insulin resistance, in which LPS-driven innate immune activation, altered SCFA production, modified bile acid signalling, and changes in microbial co-metabolites jointly mould low-grade inflammation and metabolic dysfunction [12,13,14].

### 2.2. Oxidative Stress, Redox Imbalance, and Endothelial Dysfunction

This subsection examines oxidative stress as a distinct, although interconnected, level of diabetic pathophysiology. Whereas Section 2.1 concerned the immune-metabolic drivers of insulin resistance, this section focuses on intracellular redox imbalance, hyperglycaemia-driven metabolic overflow, nitric oxide (NO) depletion, and endothelial impairment as mechanisms that transform metabolic injury into vascular injury. Oxidative stress is characterised by excessive production of reactive oxygen species (ROS), including superoxide anion, hydrogen peroxide, hydroxyl radicals, and peroxynitrite [15].

When ROS production exceeds the cellular antioxidant capacity, these reactive molecules accumulate and interact with lipids, proteins, and nucleic acids, causing structural and functional damage. In particular, lipid peroxidation affects polyunsaturated fatty acids within cellular membranes, altering membrane integrity and signalling processes.

In addition to ROS, advanced glycation end-products (AGEs) represent a major contributor to oxidative stress in diabetes. AGEs are generated through non-enzymatic glycation reactions involving proteins, lipids, and carbohydrates under conditions of chronic hyperglycaemia. Their interaction with the receptor for advanced glycation end-products (RAGE) activates intracellular signalling cascades that promote inflammation, oxidative stress, and vascular injury [16].

From a clinical perspective, however, assessing AGEs is challenging. AGEs is represented by a heterogeneous array of compounds, and circulating concentrations, tissue accumulation, and skin autofluorescence do not necessarily provide interchangeable data. Measurements may vary according to analytical method, sample handling, renal function, age, dietary intake, glycaemic exposure, oxidative status, and comorbid inflammatory conditions. Therefore, although AGEs are biologically significant markers of cumulative metabolic and oxidative harm, their routine clinical applicability is limited by a lack of standardisation, variable reproducibility, and the absence of universally validated thresholds for risk stratification or monitoring of nutrition-based interventions.

Under persistent hyperglycaemic conditions, intracellular glucose is increasingly diverted toward alternative metabolic pathways, including the polyol pathway, the hexosamine biosynthetic pathway, and the protein kinase C (PKC) signalling cascade. Although these pathways are relatively minor under physiological conditions, they become highly active in diabetes and contribute significantly to metabolic dysregulation.

The polyol pathway involves the reduction of glucose to sorbitol via aldose reductase, consuming nicotinamide adenine dinucleotide phosphate (NADPH), a key cofactor required for glutathione regeneration. This process leads to depletion of intracellular antioxidant defences and increased susceptibility to oxidative damage. Furthermore, the subsequent conversion of sorbitol to fructose increases nicotinamide adenine dinucleotide (NADH) levels, thereby disrupting cellular redox balance.

Simultaneously, excess glucose is shunted into the hexosamine pathway, leading to the production of uridine diphosphate N-acetylglucosamine (UDP-GlcNAc), which modifies proteins through O-linked glycosylation. These post-translational modifications alter the function of transcription factors and signalling proteins involved in insulin action, thereby impairing insulin sensitivity.

In parallel, chronic hyperglycaemia promotes de novo synthesis of diacylglycerol (DAG), which activates PKC isoforms. PKC activation triggers a cascade of phosphorylation events that affect endothelial permeability, gene expression, and inflammatory responses, ultimately exacerbating insulin resistance and vascular dysfunction [17].

These interconnected pathways establish a redox disequilibrium that progressively alters cellular signalling, antioxidant capacity, and tissue integrity, thereby providing the biochemical substrate for vascular and organ damage.

At the vascular level, oxidative stress promotes endothelial activation and dysfunction. Increased expression of adhesion molecules such as vascular cell adhesion molecule-1 (VCAM-1) and intercellular adhesion molecule-1 (ICAM-1) facilitates leukocyte adhesion and transmigration into the subendothelial space, where they differentiate into pro-inflammatory macrophages and contribute to atherosclerotic plaque formation [18].

Emerging evidence highlights the role of extracellular vesicles released under conditions of oxidative stress. These vesicles carry bioactive molecules, including microRNAs and proteins involved in insulin signalling, such as phosphatase and tensin homolog (PTEN), which negatively regulate the phosphatidylinositol 3-kinase–protein kinase B (PI3K–Akt) pathway. This results in reduced Akt phosphorylation and impaired glucose uptake. In addition, vesicles derived from M1 macrophages may contain microRNAs that target key regulators of insulin sensitivity, including sirtuins (SIRT) and peroxisome proliferator-activated receptors (PPAR), thereby amplifying metabolic dysfunction [19].

At the molecular level, insulin exerts vasodilatory effects by activating the PI3K–Akt pathway, leading to phosphorylation of endothelial nitric oxide synthase (eNOS) and nitric oxide (NO) production. In insulin-resistant states, this pathway is selectively impaired, whereas the mitogen-activated protein kinase (MAPK) pathway—associated with endothelin-1 production and cellular proliferation—remains relatively preserved. This imbalance promotes vasoconstriction, inflammation, and pro-atherogenic remodelling.

Mitochondrial dysfunction and increased NADPH oxidase activity further contribute to oxidative stress, leading to NO degradation and endothelial dysfunction. Human clinical studies have shown that reduced NO bioavailability is detectable even in early dysglycaemic conditions and correlates with insulin resistance, suggesting that endothelial dysfunction may lead to overt diabetes [20]. The interaction between NF-κB and peroxisome proliferator-activated receptors (PPARs) represents an additional regulatory layer connecting inflammation, lipid metabolism, and mitochondrial function. In particular, PPAR-α plays a central role in fatty acid uptake and β-oxidation, mitochondrial oxidative metabolism, and metabolic adaptation in tissues with high energy demand. In insulin-resistant and lipotoxic states, reduced PPAR-α action may promote impaired mitochondrial fatty acid handling, accumulation of lipid intermediates, increased ROS generation, and amplification of inflammatory signalling. Conversely, NF-κB activation may silence PPAR-dependent metabolic programmes, thereby reinforcing a shift from oxidative metabolism toward inflammatory and stress-responsive conditions. This reciprocal interaction suggests that the balance between NF-κB-driven inflammation and PPAR-α-mediated mitochondrial lipid oxidation may be significant for the progression from metabolic stress to endothelial dysfunction and the consequent cardiorenal injury.

Collectively, these processes make oxidative stress crucial in vascular and endothelial injury in diabetes. This framing distinguishes Section 2.2 from the cytokine-centred immune-metabolic discussion in Section 2.1 and provides the mechanistic basis for the nutrition-focused modulation discussed in Section 3.

### 2.3. Cardiorenal Organ Damage as a Shared Downstream Pathway

The pathophysiological complexity of diabetes is characterised by progressive damage to both microvascular and macrovascular structures; accordingly, it can be considered a panvascular disease. This process ultimately leads to organ dysfunction, including heart failure, chronic kidney disease (CKD), and cerebrovascular disease.

Microvascular complications predominantly affect capillaries and small arterioles and are clinically manifested as diabetic retinopathy, nephropathy, and neuropathy. These conditions arise from chronic hyperglycaemia, which induces endothelial dysfunction, thickening of the basement membrane, and impairment of perivascular support cells such as pericytes. The cumulative effect is a progressive reduction in microcirculatory perfusion and tissue oxygenation.

In contrast, macrovascular complications involve large and medium-sized arteries and include coronary artery disease (CAD), cerebrovascular disease, and peripheral arterial disease (PAD). These manifestations are primarily driven by accelerated atherosclerosis, which is strongly promoted by the chronic inflammatory and oxidative environment characteristic of diabetes. Endothelial dysfunction facilitates monocyte adhesion, migration into the intima, and subsequent differentiation into foam cells, ultimately leading to atherosclerotic plaque formation and instability.

At the myocardial level, insulin resistance induces profound metabolic alterations. Increased circulating free fatty acids, secondary to impaired insulin-mediated suppression of lipolysis, enhance myocardial fatty acid uptake through transporters such as CD36. Although initially adaptive, this metabolic shift toward fatty acid β-oxidation results in reduced metabolic efficiency, increased oxygen consumption, and excessive production of reactive oxygen species (ROS), thereby promoting mitochondrial dysfunction and oxidative stress [21].

These alterations lead to structural and functional cardiac changes, including cardiomyocyte hypertrophy, interstitial fibrosis, and adverse ventricular remodelling, which are hallmarks of diabetic cardiomyopathy. Chronic inflammation further contributes to myocardial injury by activating transforming growth factor beta (TGF-β)-mediated fibrotic pathways, ultimately impairing contractile function [22].

In the kidney, similar mechanisms contribute to the development of diabetic nephropathy. Persistent oxidative stress and inflammation promote activation of redox-sensitive signalling pathways and the NLRP3 inflammasome, resulting in increased production of pro-inflammatory cytokines such as interleukin-1β (IL-1β) and interleukin-18 (IL-18), which sustain renal inflammation and fibrosis.

At the structural level, mesangial expansion represents an early and key histopathological feature of diabetic nephropathy. This process is characterised by proliferation of mesangial cells and accumulation of extracellular matrix components, including type IV collagen, fibronectin, and laminin, ultimately leading to glomerular dysfunction and progressive decline in renal filtration capacity [23].

Renin–angiotensin–aldosterone system (RAAS) activation plays a central role in amplifying renal injury. Angiotensin II exerts not only vasoconstrictive effects but also pro-inflammatory, pro-oxidative, and pro-fibrotic actions. It stimulates mesangial cell proliferation, increases TGF-β expression, and enhances intraglomerular pressure, thereby accelerating disease progression. Pharmacological inhibition of the RAAS has consistently been shown to reduce albuminuria and slow CKD progression, underscoring the pathogenic relevance of the RAAS [24].

In addition, insulin resistance directly affects podocyte and endothelial cell function. Impaired insulin signalling disrupts cytoskeletal organisation and slit diaphragm integrity, compromising the selectivity of the glomerular filtration barrier. Hemodynamic alterations, including glomerular hyperfiltration and increased intraglomerular pressure, further exacerbate endothelial damage and promote albuminuria.

Emerging integrative models highlight the contribution of adiposity-related insulin resistance to renal injury. Lipotoxicity, mitochondrial dysfunction, and endothelial inflammation act synergistically to induce podocyte damage and mesangial expansion, thereby linking systemic metabolic dysfunction to progressive kidney disease [25].

Overall, the interplay between metabolic, inflammatory, and hemodynamic factors establishes a unified pathophysiological framework in which cardiovascular and renal complications represent parallel manifestations of a common disease continuum. This integrated perspective supports the concept of a cardio–renal–metabolic axis in diabetes [26].

**Figure 1:**
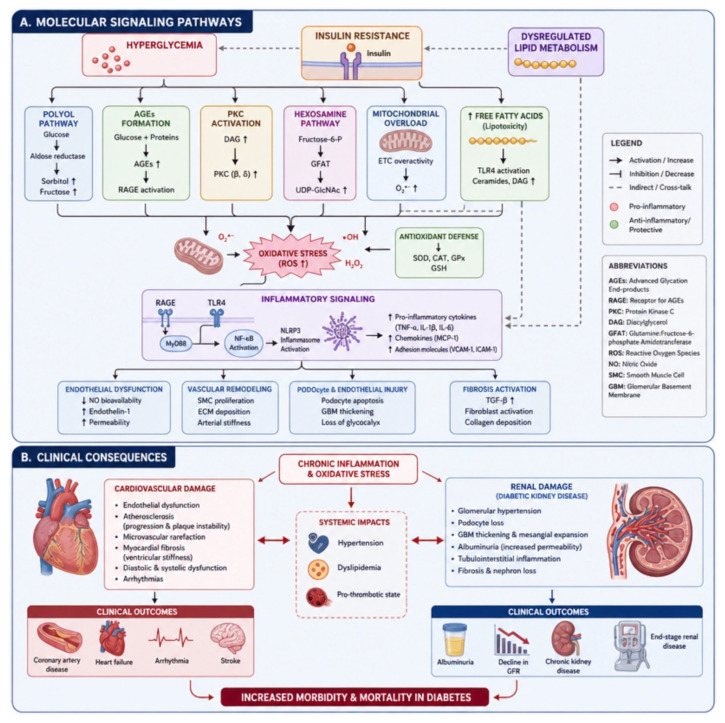
Integrated molecular signalling pathways and clinical consequences linking hyperglycaemia, insulin resistance, dysregulated lipid metabolism, inflammation, oxidative stress, and cardiorenal damage in diabetes. (**A**) Hyperglycaemia, insulin resistance, and lipid metabolic dysregulation activate multiple molecular pathways, including the polyol pathway, advanced glycation end-product formation, protein kinase C activation, the hexosamine pathway, mitochondrial overload, increased free fatty acid flux, impaired antioxidant defence, and inflammatory signalling. These mechanisms converge on oxidative stress, endothelial dysfunction, vascular remodelling, podocyte and endothelial injury, and fibrosis activation. (**B**) Chronic inflammation and oxidative stress contribute to cardiovascular damage, renal damage, systemic cardiometabolic abnormalities, and adverse clinical outcomes, ultimately increasing morbidity and mortality in diabetes. Abbreviations: AGEs: advanced glycation end-products; RAGE: receptor for advanced glycation end-products; PKC: protein kinase C; DAG: diacylglycerol; GFAT: glutamine:fructose-6-phosphate amidotransferase; UDP-GlcNAc: uridine diphosphate N-acetylglucosamine; ETC: electron transport chain; ROS: reactive oxygen species; O_2_•^−^: superoxide anion; H_2_O_2_: hydrogen peroxide; •OH: hydroxyl radical; TLR4: toll-like receptor 4; SOD: superoxide dismutase; CAT: catalase; GPx: glutathione peroxidase; GSH: reduced glutathione; MyD88: myeloid differentiation primary response 88; NF-κB: nuclear factor kappa B; NLRP3: NOD-, LRR-, and pyrin domain-containing protein 3; TNF-α: tumour necrosis factor alpha; IL-1β: interleukin-1 beta; IL-6: interleukin-6; MCP-1: monocyte chemoattractant protein-1; VCAM-1: vascular cell adhesion molecule-1; ICAM-1: intercellular adhesion molecule-1; NO: nitric oxide; SMC: smooth muscle cell; ECM: extracellular matrix; GBM: glomerular basement membrane; TGF-β: transforming growth factor beta; GFR: glomerular filtration rate.

These integrated inflammatory, oxidative, metabolic, and cardiorenal pathways are summarised in Figure 1.

### 2.4. Genetic and Epigenetic Mechanisms: Molecular Foundations of Metabolic Dysfunction

Genome-wide association studies have identified multiple genetic loci associated with insulin resistance and type 2 diabetes mellitus (T2DM). However, these loci explain only a limited proportion of disease heritability, giving rise to the concept of “missing heritability,” which may be partially accounted for by epigenetic mechanisms.

Epigenetics refers to heritable changes in gene expression that occur without alterations in the underlying DNA sequence. Among these, DNA methylation—the addition of a methyl group to cytosine residues within CpG dinucleotides—has emerged as a key regulatory mechanism that modulates transcriptional activity. In the context of metabolic disease, epigenetic modifications represent a critical interface between genetic predisposition and environmental factors, such as diet, oxidative stress, obesity, and chronic inflammation [27].

In addition to DNA methylation, post-translational histone modifications and non-coding RNAs—including microRNAs and long non-coding RNAs—contribute to the regulation of genes involved in inflammation, glucose metabolism, and insulin signalling. Dysregulation of these mechanisms has been implicated in β-cell dysfunction and impaired insulin responsiveness.

Dietary exposures may modulate this epigenetic interface through several convergent processes. Nutrients implicated in one-carbon metabolism, including folate, choline, betaine, and B vitamins, can influence methyl-group availability for DNA methylation, whereas fatty acid quality, polyphenols, fibre-derived short-chain fatty acids, and microbiota-derived metabolites may modulate histone acetylation, histone deacetylase activity, and non-coding RNA expression. Through these mechanisms, diet may regulate the transcription of genes involved in inflammatory and metabolic pathways, including NF-κB-related cytokine signalling, oxidative stress responses, mitochondrial function, and insulin signalling [27].

Aberrant methylation patterns affecting key metabolic genes, such as pancreatic and duodenal homeobox 1 (PDX1) and glucagon-like peptide-1 receptor (GLP-1R), have been associated with reduced insulin secretion and β-cell dysfunction. Similarly, increased expression of histone deacetylases and dysregulation of microRNAs—including miR-21 and miR-146a—as well as long non-coding RNAs such as metastasis-associated lung adenocarcinoma transcript 1 (MALAT1) contribute to the development of insulin resistance and metabolic impairment [27].

Furthermore, epigenetic mechanisms may mediate “metabolic memory,” whereby early-life environmental exposures induce long-lasting changes in gene expression that influence disease susceptibility later in life. This concept is supported by evidence demonstrating that adverse intrauterine or early postnatal environments can predispose individuals to insulin resistance and T2DM in adulthood.

Genome-wide methylation studies have identified consistent alterations in genes involved in glucose and lipid metabolism, including peroxisome proliferator-activated receptor gamma coactivator 1 alpha (PPARGC1A) and ATP-binding cassette transporter G1 (ABCG1). These changes have been observed across multiple tissues—including blood, adipose tissue, skeletal muscle, and pancreatic islets—suggesting a systemic epigenetic reprogramming associated with metabolic disease [28].

In skeletal muscle, aberrant methylation of genes regulating mitochondrial function and oxidative metabolism contributes to impaired insulin-stimulated glucose uptake. In pancreatic β-cells, epigenetic dysregulation interacts with oxidative stress to compromise insulin secretion and promote cellular dysfunction.

Importantly, epigenetic modifications may precede the clinical onset of disease and therefore represent potential predictive biomarkers. Prospective human cohort studies have shown that specific DNA methylation signatures are linked to future risk of hyperglycaemia and T2DM, even after adjustment for traditional risk factors [28].

The interaction between genetic and epigenetic factors also contributes to the heterogeneity observed in metabolic disease. In particular, obesity-related metabolic stress can induce widespread epigenomic remodelling, yet individual responses vary significantly depending on underlying genetic susceptibility.

Emerging evidence suggests that lifestyle interventions, including weight loss, physical activity, and dietary modification, as well as pharmacological therapies, can partially reverse epigenetic alterations. From a nutritional perspective, this implies that dietary patterns may play a role not only through short-term effects on glycaemia, lipids, and oxidative stress, but also by influencing inflammatory gene-expression programmes. Diets rich in fibre, unsaturated fatty acids, polyphenols, and micronutrients may promote a less-inflammatory epigenetic profile, whereas excess refined carbohydrates, saturated fats, and ultra-processed foods may promote epigenetic signatures correlated with low-grade inflammation, insulin resistance, and metabolic memory. However, the long-term stability and clinical relevance of these diet-related epigenetic changes remain to be fully elucidated [29].

Overall, the importance of epigenetics in T2DM lies in its ability to link environmental exposures, including nutrition, to sustained inflammatory and metabolic reprogramming. This supports the idea that precision nutrition should also account for epigenetic susceptibility and diet-responsive molecular signatures, while recognising that epigenetic biomarkers are not yet useful for routine clinical decision-making [27,28,29].

### 2.5. Obesity as an Amplifier of Metabolic Inflammation

Obesity increases the inflammatory and oxidative pathways involved in type 2 diabetes mellitus. Expansion of visceral adipose tissue leads to adipocyte hypertrophy, local hypoxia, mitochondrial stress, and immune-cell recruitment, thereby promoting a shift toward a pro-inflammatory adipose tissue phenotype. In this context, increased infiltration of M1-like macrophages and altered adipokine release supports chronic low-grade inflammation, impaired insulin signalling, and systemic metabolic impairment [9].

Beyond adipose tissue inflammation, obesity encourages ectopic lipid accumulation in insulin-sensitive organs, including the liver, skeletal muscle, heart, and kidney. Increased free fatty acid flux, lipotoxic intermediates, mitochondrial dysfunction, and oxidative stress collectively affect insulin action and result in endothelial dysfunction, diabetic cardiomyopathy, and renal injury [21,25]. Therefore, obesity should not be considered only as an excess of body weight, but as a biological condition characterised by adipose tissue dysfunction, lipotoxicity, redox imbalance, and cardiorenal vulnerability.

Obesity is also closely associated with alterations in the gut microbiota and intestinal barrier dysfunction. Dysbiosis may favour metabolic endotoxemia and modify short-chain fatty acid production, bile acid signalling, and other microbial co-metabolites, causing further inflammatory tone and insulin resistance [12,13,14]. These processes provide a strong rationale for nutritional strategies aimed not only at weight reduction, but also at improving dietary quality, adipose tissue function, gut microbial ecology, oxidative balance, and long-term cardiometabolic resilience.

## 3. Nutritional Modulation of Inflammation and Oxidative Stress

### 3.1. Macronutrient Quality and Metabolic Inflammation

This subsection shifts from disease mechanisms to dietary exposure as a modifiable upstream factor. Its aim is not to re-describe the ROS–cytokine loops outlined in Section 2.1 and Section 2.2, but to highlight how the quality of carbohydrate, fat, and protein intake acutely or chronically amplifies—or attenuates—those pathways. Evidence from acute nutritional challenge studies in humans suggests that isocaloric loads of glucose, lipids, and proteins are not metabolically equivalent with respect to postprandial redox signalling, leukocyte activation, and endothelial stress.

Glucose and lipid loads have been shown to induce a rapid increase in ROS production by circulating leukocytes, whereas protein intake elicits a comparatively lower oxidative response [30,31]. In this nutritional context, activation of NADPH oxidase, upregulation of p47^phox, NF-κB activation, and silencing IκBα should be interpreted as postprandial amplification signals rather than as a separate reiteration of the basal inflammatory processes described above.

Refined carbohydrate exposure may also promote a transient prothrombotic and endothelial-activating phenotype. Hyperglycaemia activates transcription factors such as activator protein-1 (AP-1) and early growth response-1 (EGR-1), increasing expression of tissue factor, plasminogen activator inhibitor-1 (PAI-1), and matrix metalloproteinases (MMP-2 and MMP-9). These effects link meal composition to vascular risk through acute functional changes rather than through chronic cytokine burden alone [32,33].

Saturated fat-rich meals induce a similarly adverse nutritional signal. High-fat or mixed high-fat/high-carbohydrate meals increase lipid peroxidation, endotoxemia, and monocyte activation; these effects involve toll-like receptor 4 (TLR4)/CD14 signalling, suppressor of cytokine signalling-3 (SOCS-3), adhesion molecule expression, and markers of macrophage activation such as CD68 and platelet endothelial cell adhesion molecule (PECAM) [34,35,36].

The relevance of these postprandial responses lies in their capacity to interfere with insulin signalling. For example, TNF-α can reduce insulin receptor tyrosine phosphorylation, whereas SOCS-3 promotes ubiquitination and degradation of insulin receptor substrate-1 (IRS-1). In Section 3.1, these mediators are therefore considered as diet-responsive readouts of metabolic inflammation rather than as a renewed description of the cytokine network itself.

Macronutrient quality is therefore best described as a determinant of pathway intensity and timing. Refined carbohydrates and saturated fats tend to amplify postprandial oxidative and inflammatory signalling, whereas protein-dominant loads and balanced macronutrient profiles appear to induce a lower acute redox response. This distinction adds a nutritional layer to the pathophysiological framework presented in Section 2.

Collectively, these findings emphasise that both the quantity and the quality of macronutrients modulate metabolic inflammation through their effects on postprandial oxidative stress, endotoxemia, leukocyte activation, insulin signalling, and endothelial function. This provides the rationale for moving from single-nutrient effects to broader dietary patterns, as discussed in Section 3.2.

### 3.2. Dietary Patterns: Mediterranean, Dash, and Plant-Based Models

Beyond individual nutrients, whole dietary patterns exert a coordinated influence on inflammatory and oxidative pathways through their combined effects on macronutrient composition, micronutrient density, fiber content, fatty acid profile, and gut microbiota modulation.

The Mediterranean diet is the most studied dietary regimen in this context. Characterised by high consumption of vegetables, legumes, whole grains, fruits, nuts, and extra-virgin olive oil, along with moderate intake of fish and a low one of red and processed meats, this dietary regimen supplies monounsaturated fatty acids, *n*-3 PUFAs—including ALA from plant sources and EPA/DHA from fish and seafood—, polyphenols, and fermentable fibre. Adherence to the Mediterranean diet has been consistently associated with reduced circulating levels of inflammatory biomarkers, including interleukin-1β (IL-1β), interleukin-6 (IL-6), tumour necrosis factor alpha (TNF-α), monocyte chemoattractant protein-1 (MCP-1), and vascular cell adhesion molecule-1 (VCAM-1), as well as with improved endothelial function and oxidative balance [37,38].

At the molecular level, these effects are mediated through attenuation of NF-κB signalling, enhancement of nitric oxide (NO) bioavailability, activation of AMP-activated protein kinase (AMPK) and sirtuin 1 (SIRT1), and modulation of gut microbiota composition, leading to reduced metabolic endotoxemia and improved insulin sensitivity [39,40].

Clinical evidence from human studies further suggests that the Mediterranean diet improves glycaemic control, reduces cardiovascular risk factors, and promotes weight loss in patients with type 2 diabetes mellitus (T2DM), thereby reinforcing its relevance as a therapeutic dietary strategy [41].

The Dietary Approaches to Stop Hypertension (DASH) diet shares several beneficial characteristics with the Mediterranean diet, including high intake of fruits, vegetables, whole grains, legumes, and low-fat dairy products, combined with reduced sodium and red meat consumption. Although originally developed for blood pressure control, the DASH diet has demonstrated broader metabolic benefits.

Specifically, adherence to the DASH diet has been associated with reductions in systolic and diastolic blood pressure, improvements in insulin sensitivity, and reductions in total cholesterol and fasting insulin levels [42,43]. These effects are likely mediated by its high content of potassium, magnesium, fibre, and antioxidants, which collectively contribute to improved endothelial function and reduced oxidative stress.

Plant-based dietary patterns, including vegetarian and vegan diets, further extend these benefits. These dietary models are typically characterised by high intake of fibre, phytochemicals, and unsaturated fats, along with low intake of saturated fats, heme iron, and advanced glycation end-products (AGEs). Human observational and interventional studies report that plant-based diets are correlated with lower levels of C-reactive protein (CRP) and IL-6, reflecting a reduced inflammatory burden [44].

In contrast, Western dietary patterns—characterised by high consumption of refined carbohydrates, ultra-processed foods, red meat, and saturated fats—are strongly associated with increased oxidative stress, chronic inflammation, and gut microbiota dysbiosis. These dietary exposures promote metabolic endotoxemia and activation of inflammatory pathways, thereby contributing to the development and progression of insulin resistance and T2DM [45].

Collectively, these findings highlight that dietary patterns represent a key determinant of metabolic health, acting through integrated effects on inflammation, oxidative stress, endothelial function, and gut microbiota. This supports a shift from a nutrient-centric to a pattern-based approach in nutritional strategies for diabetes management.

### 3.3. Fatty Acids, Dietary Fibre, Gut Microbiota, and Redox Balance

**Figure 2:**
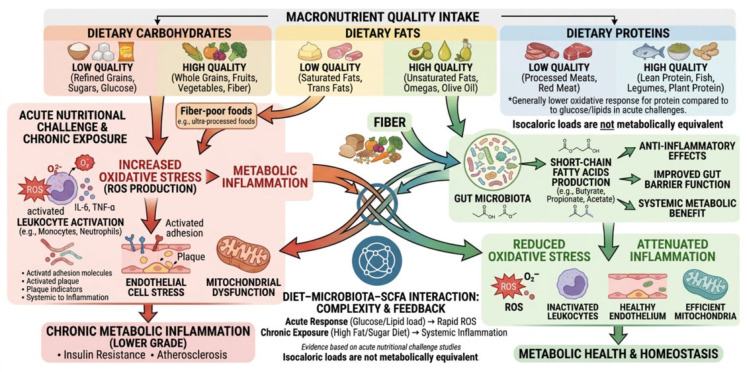
Nutritional modulation of metabolic inflammation through macronutrient quality and diet–microbiota–short-chain fatty acid interactions. Low-quality carbohydrates and fats, particularly refined sugars, low-fibre foods, saturated fats, and trans fats, promote postprandial oxidative stress, leukocyte activation, endothelial cell stress, mitochondrial dysfunction, and low-grade metabolic inflammation. Conversely, high-quality carbohydrate and fat sources, such as whole grains, fruits, vegetables, dietary fibre, unsaturated fatty acids, including omega-3 polyunsaturated fatty acids from plant and marine sources, and olive oil, promote gut microbiota homeostasis and short-chain fatty acid production. By influencing intestinal barrier integrity, endotoxemia, inflammatory signalling, redox balance, and insulin sensitivity, the diet–microbiota–short-chain fatty acid axis links dietary patterns to downstream cardiometabolic risk. Abbreviations: SCFA: short-chain fatty acid; ROS: reactive oxygen species; O_2_•^−^: superoxide anion; IL-6: interleukin-6; TNF-α: tumour necrosis factor alpha.

The inflammatory and oxidative effects of diet are determined not only by total caloric intake but also by the biochemical quality of macronutrients and their interaction with the gut microbiota. In particular, the type of dietary fatty acids and the presence of fermentable substrates such as dietary fibre play a central role in modulating metabolic inflammation and redox balance. The main diet–microbiota–SCFA interactions and their downstream metabolic results are summarised in Figure 2.

Saturated fatty acids (SFAs), particularly palmitate, have been shown to promote inflammation by activating NF-κB-dependent pathways and increasing the production of pro-inflammatory cytokines such as interleukin-6 (IL-6). In addition, SFAs contribute to mitochondrial dysfunction and increased reactive oxygen species (ROS) production, leading to a more oxidised intracellular environment characterised by reduced glutathione (GSH) availability and impaired antioxidant capacity [46,47].

On the other hand, unsaturated fatty acids have guarding metabolic effects depending on their class and dietary source. *n*-3 PUFAs include ALA, mainly provided by plant sources such as seeds, nuts, and selected vegetable oils, and EPA and DHA, mainly derived from fish and seafood. *n*-6 PUFAs represent a distinct PUFA class and should not be conflated with *n*-3 PUFAs. Diets enriched in *n*-6 PUFAs, compared with saturated fats, have been linked to reduced hepatic fat accumulation, increased insulin sensitivity, and modulation of lipid metabolism, including lower circulating levels of pro-atherogenic lipoproteins [48].

Dietary fibre represents a key mediator linking nutrition, gut microbiota, and host metabolism. In acute human intervention studies, adding fibre to high-fat, high-calorie meals attenuated ROS generation, reduced endotoxemia, and suppressed activation of toll-like receptor (TLR) pathways, leading to decreased expression of inflammatory mediators such as tumour necrosis factor alpha (TNF-α) and interleukin-1β (IL-1β) [49].

The beneficial effects of dietary fibre are largely mediated through its fermentation by gut microbiota, leading to the production of short-chain fatty acids (SCFAs), including acetate, propionate, and butyrate. These metabolites act as signalling molecules via free fatty acid receptors (FFAR2 and FFAR3) and stimulate the release of gut hormones such as glucagon-like peptide-1 (GLP-1) and peptide YY (PYY), thereby improving insulin secretion, regulating appetite, and modulating energy balance [50,51].

Beyond metabolic regulation, SCFAs play a crucial role in maintaining intestinal barrier integrity and reducing systemic inflammation. They have been shown to enhance tight junction protein expression, limit lipopolysaccharide (LPS) translocation, and attenuate oxidative stress by modulating antioxidant enzyme systems [52].

Within this context, β-glucans—soluble fibres derived from cereals, yeast, and fungi—have attracted increasing attention. Cereal β-glucans improve glycaemic control by increasing intestinal viscosity, delaying glucose absorption, enhancing bile acid excretion, and promoting low-density lipoprotein (LDL) clearance through upregulation of cholesterol metabolism pathways.

In addition, microbial-derived β-glucans interact with immune receptors such as Dectin-1, complement receptor 3 (CR3), and toll-like receptors, influencing innate immune responses and inflammatory signalling. Experimental studies have demonstrated that β-glucans modulate key metabolic pathways, including AMP-activated protein kinase (AMPK), phosphatidylinositol 3-kinase–protein kinase B (PI3K–Akt), and nuclear factor erythroid 2–related factor 2 (Nrf2)/heme oxygenase-1 (HO-1), ultimately improving insulin sensitivity and reducing oxidative stress [53].

Overall, these findings underscore the critical roles of nutrient quality and microbiota-derived metabolites in shaping the inflammatory and redox environment, highlighting the importance of dietary strategies to promote beneficial microbial composition and metabolic resilience.

### 3.4. Polyphenols, Micronutrients, and Activation of Antioxidant Signalling

Polyphenol-rich foods represent a key link between dietary quality and the activation of endogenous antioxidant defence systems. Beyond their direct radical-scavenging properties, polyphenols exert their biological effects primarily by modulating intracellular signalling pathways involved in oxidative stress and inflammation.

In the postprandial state, dietary supplementation with polyphenol-rich compounds, such as resveratrol and grape-derived polyphenols, has been shown to attenuate meal-induced oxidative stress and inflammatory responses. These effects include suppression of NF-κB activation, reduction in reactive oxygen species (ROS) generation, and preservation of antioxidant enzyme expression [54].

A crucial mechanism underlying these effects includes the activation of the nuclear factor erythroid 2–related factor 2 (Nrf2) pathway. Nrf2 plays as a master regulator of the antioxidant response by encouraging the transcription of genes involved in detoxification, glutathione metabolism, and cytoprotection, including heme oxygenase-1 (HO-1), NAD(P)H quinone dehydrogenase 1 (NQO1), and glutathione S-transferases. Beyond antioxidant defence, Nrf2 activation may decrease inflammatory responses by limiting redox-sensitive NF-κB activation, reducing oxidative amplification of cytokine signalling, and preserving mitochondrial and endothelial cell integrity. In this context, activation of the Nrf2–Keap1/ARE pathway may reduce lipid peroxidation, protein oxidation, mitochondrial stress, and cumulative cellular injury. Polyphenols have been shown to prevent the downregulation of Nrf2 induced by high-fat, high-carbohydrate meals, thereby restoring redox balance, reducing inflammatory activation, and increasing cellular resilience to oxidative injury [54,55].

This paradigm highlights that effective nutritional strategies should not be limited to exogenous antioxidant supplementation but should instead focus on activating endogenous cytoprotective pathways. In this context, the Nrf2–Kelch-like ECH-associated protein 1 (Keap1) axis represents a critical molecular target linking diet to cellular defence mechanisms.

The anti-inflammatory effects of polyphenol-rich foods are further supported by acute intervention studies. For instance, consumption of orange juice, despite its carbohydrate content, has been shown to prevent postprandial increases in endotoxemia, toll-like receptor 4 (TLR4) expression, ROS generation, and NF-κB activation, likely due to the presence of flavanones such as hesperidin and naringenin [15].

Micronutrients also have a central role in redox homeostasis. Vitamin C acts as a water-soluble antioxidant, promoting the neutralisation of reactive oxygen species, regenerating other antioxidants, and maintaining endothelial nitric oxide bioavailability. Vitamin E, as a lipid-soluble antioxidant, prevents cell membranes and lipoproteins from lipid peroxidation. Selenium is requested for the activity of glutathione peroxidases and other selenoproteins involved in peroxide detoxification, whereas zinc takes part in antioxidant defence, partly through its role in superoxide dismutase activity and influencing inflammatory signalling. Magnesium may further support metabolic homeostasis by modulating insulin signalling, vascular tone, and oxidative stress responses.

Importantly, the biological efficacy of micronutrients is strongly influenced by their dietary context. Whole-food sources provide a complex matrix of bioactive compounds that interact synergistically to modulate oxidative stress and inflammation. In contrast, isolated supplementation may not reproduce the same beneficial effects, highlighting the importance of dietary patterns over single-nutrient approaches.

Overall, the available evidence supports a shift toward dietary strategies that enhance endogenous antioxidant capacity, reduce oxidative stress, and modulate inflammatory pathways through integrated effects on cellular signalling networks. The main nutrients and dietary patterns involved in the modulation of inflammatory and oxidative pathways in diabetes are summarised in Table 1.

## 4. Impact of Nutrition on Diabetic Cardiovascular and Renal Complications

### 4.1. Nutritional Effects on Endothelial Function and Atherosclerosis

Dietary patterns exert a profound influence on endothelial function and the development of atherosclerosis, primarily by modulating oxidative stress, inflammation, and lipid metabolism.

The Mediterranean diet is characterised by high intake of monounsaturated fatty acids—particularly oleic acid from olive oil—along with polyphenols such as hydroxytyrosol and *n*-3 polyunsaturated fatty acids. These components enhance mitochondrial efficiency, reduce electron leakage, and limit reactive oxygen species (ROS) production, thereby improving endothelial function.

A meta-analysis of randomised controlled trials in humans showed that adherence to a Mediterranean diet significantly increases flow-mediated dilation (FMD), a key marker of endothelial function, in both healthy subjects and patients with increased cardiovascular risk [56].

In addition to endothelial effects, lipid mediators such as ceramides play an important role in vascular health. Elevated ceramide levels promote apoptosis and impair insulin signalling, contributing to endothelial dysfunction. Adherence to the Mediterranean diet has been associated with reductions in low-density lipoprotein (LDL) cholesterol and circulating ceramides, as well as increased levels of adiponectin, a vasculoprotective adipokine [57].

In contrast, diets rich in saturated fatty acids promote a pro-atherogenic phenotype. Saturated fats activate toll-like receptor 4 (TLR4), leading to NF-κB activation and increased production of pro-inflammatory cytokines such as interleukin-6 (IL-6) and adhesion molecules. This process enhances leukocyte adhesion, promotes LDL oxidation, and facilitates foam cell formation within the vascular wall, representing an early step in atherogenesis.

The Dietary Approaches to Stop Hypertension (DASH) diet exerts similar beneficial effects. Its high levels of potassium, magnesium, and antioxidants improve endothelial function by enhancing nitric oxide (NO) bioavailability and reducing calcium overload in vascular smooth muscle cells. These mechanisms promote vasodilation, inhibit platelet aggregation, and reduce vascular inflammation.

Human clinical studies have reported that adherence to the DASH diet significantly reduces systolic and diastolic blood pressure, improves natriuresis, and lowers vascular resistance, thereby reducing cardiovascular risk [58].

Overall, these findings support the concept that dietary patterns modulate endothelial function through integrated effects on oxidative stress, inflammatory signalling, lipid metabolism, and vascular homeostasis.

### 4.2. Role of Dietary Protein, Sodium, and Metabolic Load in Diabetic Kidney Disease

Chronic kidney disease (CKD) is one of the most common and clinically relevant complications of diabetes mellitus. Nutritional factors play a central role in both the development and progression of diabetic kidney disease (DKD), particularly by modulating protein intake, sodium balance, and overall metabolic load.

Current guidelines recommend a dietary protein intake of approximately 0.8 g/kg/day in patients with CKD. Both excessive and insufficient protein intake may be detrimental: high protein consumption can increase intraglomerular pressure and accelerate renal damage, whereas inadequate intake may lead to malnutrition and adverse clinical outcomes [59].

Sodium intake represents another critical factor. A daily intake below 2 g is generally recommended in patients with CKD, particularly in those with diabetes. Excess sodium intake contributes to fluid retention, hypertension, and activation of the renin–angiotensin–aldosterone system (RAAS), thereby promoting renal injury and cardiovascular complications.

In addition, chronic hyperglycaemia and excessive carbohydrate intake contribute to renal damage through the formation of advanced glycation end-products (AGEs), which induce oxidative stress and inflammation by activating NF-κB signalling pathways. These processes increase glomerular permeability and promote albuminuria, a key marker of kidney disease progression.

Recent human cohort studies indicate that moderate carbohydrate intake is correlated with lower mortality than both high and very low carbohydrate intake, highlighting the relevance of dietary balance over extreme restriction. In particular, diets rich in complex carbohydrates appear to exert more favourable metabolic effects than those high in simple sugars, which are associated with hyperglycaemia, insulin resistance, and increased lipogenesis [60].

Excessive intake of refined carbohydrates also contributes to systemic inflammation, oxidative stress, and endothelial dysfunction, thereby accelerating the progression of CKD. These findings emphasise the importance of dietary quality in addition to quantity.

Assessment of sodium and protein intake remains challenging in clinical practice. Although 24 h urinary excretion represents the most accurate method, it is limited by variability related to physical activity, muscle metabolism, and individual differences in renal handling of electrolytes.

Overall, current evidence supports a dietary approach characterised by moderate protein intake, reduced sodium consumption, and balanced carbohydrate quality as a strategy to slow CKD progression and improve clinical outcomes in patients with diabetes. However, further long-term studies are needed to refine dietary recommendations and support personalised nutritional interventions.

**Figure 3:**
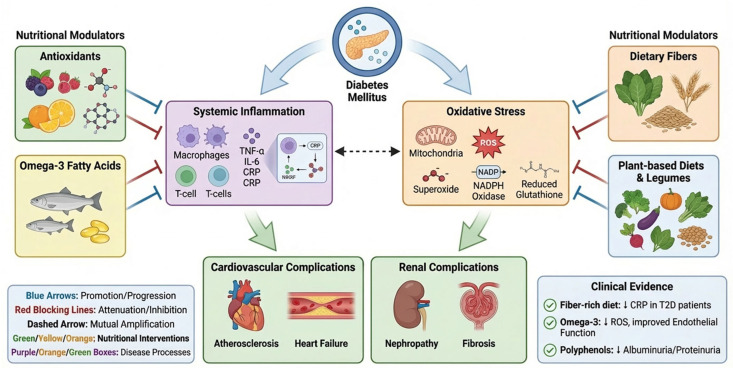
Nutrition-mediated modulation of inflammation and oxidative stress across the cardiovascular–renal continuum in diabetes. TNF-α: tumour necrosis factor alpha; IL-6: interleukin-6; CRP: C-reactive protein; ROS: reactive oxygen species; NADP: nicotinamide adenine dinucleotide phosphate; NADPH: reduced nicotinamide adenine dinucleotide phosphate; T2D: type 2 diabetes; Nrf2: nuclear factor erythroid 2-related factor 2. Blue and red inhibitory lines ending with a T-bar indicate attenuation or inhibition of the corresponding pathological pathways by specific nutritional modulators. Different colors are used only to visually distinguish separate inhibitory connections and do not indicate different biological categories.

The integrated pathways linking nutrition-mediated modulation of inflammation and oxidative stress to cardiovascular and renal consequences in diabetes are summarised in Figure 3.

## 5. Biomarkers of Inflammation and Oxidative Stress in Nutritional Studies

### 5.1. Established Inflammatory and Oxidative Biomarkers

Biomarkers play a crucial role in translating mechanistic insights from nutritional research into clinically meaningful outcomes. In the context of diabetes, inflammatory and oxidative stress biomarkers are widely used to evaluate the impact of dietary interventions on metabolic and vascular health.

Among inflammatory markers, circulating cytokines such as interleukin-6 (IL-6), tumour necrosis factor-α (TNF-α), and interleukin-1β (IL-1β) are key mediators of low-grade chronic inflammation and are closely associated with insulin resistance, endothelial dysfunction, and β-cell stress. However, their clinical utility is limited by short half-life, significant biological variability, and sensitivity to circadian and postprandial fluctuations [61].

For these reasons, acute-phase proteins—particularly C-reactive protein (CRP) and serum amyloid A (SAA)—are more frequently used in clinical and interventional studies. These markers provide a more stable measure of systemic inflammation, although they lack specificity for the underlying molecular pathways [61,62].

Oxidative stress biomarkers include indices of lipid, protein, and DNA damage. Malondialdehyde (MDA) and thiobarbituric acid reactive substances (TBARS) are commonly used to assess lipid peroxidation, although their specificity is limited. More robust markers include F2-isoprostanes, which are considered the gold standard for assessing systemic oxidative stress, despite the complexity and cost of measurement [61,63].

Markers of DNA oxidation, such as 8-hydroxy-2′-deoxyguanosine (8-OHdG), and protein oxidation, including protein carbonyls, provide additional insight into cumulative oxidative damage. These biomarkers are particularly useful in chronic disease settings but are influenced by multiple physiological and methodological factors.

In parallel, endogenous antioxidant systems can be assessed through measurement of enzymatic activity, including superoxide dismutase (SOD), catalase, and glutathione peroxidase (GPx), as well as the ratio between reduced and oxidised glutathione (GSH/GSSG), which reflects intracellular redox balance [5].

Collectively, these biomarkers provide complementary information on inflammatory and oxidative processes, although their interpretation requires careful consideration of biological variability, methodological limitations, and study design.

### 5.2. Emerging Biomarkers and Nutritional Modulation

In recent years, increasing attention has been directed toward emerging biomarkers that provide more specific insights into the interplay between diet, metabolism, and inflammation.

Myeloperoxidase (MPO), an enzyme released by activated neutrophils, represents a marker of oxidative and inflammatory activity and has been associated with endothelial dysfunction and cardiovascular risk. Similarly, nitrotyrosine reflects nitrosative stress and serves as a marker of protein modification induced by reactive nitrogen species [61].

Lipid peroxidation products such as 4-hydroxynonenal (4-HNE) not only serve as markers of oxidative damage but also act as signalling molecules that modulate inflammatory pathways and cellular stress responses.

The gut microbiota has also emerged as a key source of metabolically relevant biomarkers. Short-chain fatty acids (SCFAs), including acetate, propionate, and butyrate, reflect microbial fermentation of dietary fibre and are associated with improved metabolic and inflammatory profiles. These metabolites play a central role in linking dietary patterns to host metabolic responses [64].

Conversely, markers of metabolic endotoxemia, such as circulating lipopolysaccharide (LPS), indicate increased intestinal permeability and activation of inflammatory pathways. Elevated LPS levels have been associated with insulin resistance, obesity, and diabetes progression [65].

Another emerging biomarker is trimethylamine N-oxide (TMAO), a metabolite derived from gut microbial metabolism of dietary nutrients such as choline and carnitine. TMAO has been associated with cardiovascular risk and may reflect diet–microbiota interactions, although its interpretation is influenced by multiple confounding factors, including renal function and dietary composition [64].

These emerging biomarkers offer valuable insights into the complex relationship among diet, microbiota, and metabolic health, supporting a more integrated, systems-level understanding of nutritional interventions.

### 5.3. Challenges and Future Directions

Despite significant advances, several challenges limit the clinical applicability of inflammatory and oxidative stress biomarkers in nutritional research.

First, substantial inter-individual variability—driven by genetic, metabolic, and environmental factors—complicates the interpretation of biomarker responses to dietary interventions. In addition, pre-analytical and analytical variability, including sample collection, processing, and assay methods, can significantly influence results.

Second, many biomarkers are influenced by confounding factors such as obesity, smoking, pharmacological treatments, renal function, and timing of sample collection. These factors may obscure true diet-related effects and limit comparability across studies.

Third, the lack of standardised panels and validated thresholds for clinical interpretation hampers the translation of biomarker data into routine practice. Even promising candidates often fail to demonstrate sufficient reproducibility or specificity to support widespread clinical use.

Future research should focus on developing multi-marker panels that integrate inflammatory, oxidative, metabolic, and microbiota-derived indicators. The application of high-throughput technologies—including metabolomics, proteomics, and transcriptomics—may enable identification of novel biomarkers and improve mechanistic understanding of diet–disease interactions.

In this context, machine learning approaches offer promising opportunities to integrate complex datasets and identify predictive biomarker signatures associated with dietary patterns and clinical outcomes. Such approaches may facilitate the transition toward precision nutrition strategies tailored to individual metabolic profiles. The established and emerging biomarkers of inflammation and oxidative stress used in nutrition-based intervention studies are summarised in Table 2.

## 6. Evidence from Clinical and Interventional Studies

### 6.1. Effects of Dietary Interventions on Inflammatory and Oxidative Biomarkers

Chronic low-grade inflammation represents a central pathophysiological mechanism linking overweight and obesity to an increased risk of cardiovascular disease, type 2 diabetes mellitus (T2DM), certain cancers, and all-cause mortality. This persistent inflammatory state is closely associated with enhanced oxidative stress, both of which contribute to the development of metabolic dysfunction and progressive organ damage.

Chronic low-grade inflammation is strongly associated with visceral adipose tissue expansion, which promotes adipocyte hypertrophy, local hypoxia, and the recruitment of pro-inflammatory immune cells, particularly M1 macrophages. This process results in increased circulating levels of inflammatory mediators, including tumour necrosis factor-α (TNF-α), interleukin-6 (IL-6), and C-reactive protein (CRP), which play a central role in the development of insulin resistance, endothelial dysfunction, and hepatic steatosis.

Concurrently, alterations in gut microbiota composition and impaired intestinal barrier integrity may lead to metabolic endotoxemia. In this condition, translocation of lipopolysaccharides (LPS) activates Toll-like receptor 4 (TLR4), thereby amplifying systemic inflammatory signalling and contributing to increased cardiometabolic risk [66,67].

In this context, diet emerges as a key modulator of oxidative stress, influencing both the production of reactive oxygen species and the efficiency of antioxidant defences. Increasing evidence highlights the role of the gut microbiota as an intermediary in this process, mediating the effects of dietary components on host immune responses and redox homeostasis, thereby contributing to the regulation of inflammation and metabolic health [68,69].

A range of dietary patterns has been proposed to manage metabolic risk, particularly in T2DM. Among these, the Mediterranean diet, low-carbohydrate regimens, vegetarian or plant-based approaches, the Dietary Approaches to Stop Hypertension (DASH) diet, and macrobiotic diets have consistently demonstrated beneficial effects in individuals with T2DM.

In contrast, evidence supporting other dietary strategies—such as the Palaeolithic diet, very-low-fat diets, and intermittent fasting—remains less consistent and requires further validation. A meta-analysis of seven studies and 338 patients with T2DM reported that intermittent fasting was associated with greater weight loss but did not result in additional improvements in glycated haemoglobin (HbA1c) levels compared with standard dietary interventions [70].

Extra virgin olive oil (EVOO) is composed predominantly of triglycerides (approximately 97–99%), while a minor fraction (1–3%) consists of bioactive compounds that largely account for its biological properties. It is particularly rich in monounsaturated fatty acids (MUFAs) and also contains smaller amounts of polyunsaturated fatty acids (PUFAs).

A substantial body of evidence indicates that EVOO exerts beneficial effects on key clinical biomarkers, including reductions in blood pressure and low-density lipoprotein (LDL) cholesterol, increases in high-density lipoprotein (HDL) cholesterol, and improvements in glycaemic control and body weight regulation. Its anti-inflammatory effects are particularly relevant: high-oleocanthal EVOO has been shown to reduce pro-inflammatory cytokines—including IL-6, IL-17A, TNF-α, and IL-1β—while increasing the anti-inflammatory cytokine IL-10 [71,72].

### 6.2. Impact on Cardiovascular and Renal Clinical Outcomes

Dietary patterns exert a substantial impact on cardiovascular and renal clinical outcomes. Evidence-based dietary models such as the Mediterranean diet, DASH diet, and plant-based diets are consistently associated with reduced cardiovascular disease risk, lower mortality, and slower progression of chronic kidney disease (CKD).

Key cardioprotective dietary components include fruits, vegetables, whole grains, legumes, nuts, fish, and unsaturated fats—particularly olive oil—whereas harmful components include saturated and trans fats, refined carbohydrates, added sugars, high sodium intake, ultra-processed foods, and processed meats [73].

High adherence to healthy dietary patterns has been associated with approximately a 20% reduction in cardiovascular disease mortality [74]. The Mediterranean diet enriched with extra virgin olive oil reduced major cardiovascular events by 31% in the PREDIMED trial and by approximately 22–25% in the CORDIOPREV trial [39,75].

Behavioural interventions promoting healthy dietary habits and physical activity have also demonstrated a relative risk reduction of approximately 20% in composite cardiovascular outcomes.

According to current evidence, individuals with CKD are encouraged to adopt dietary patterns characterised by higher intake of plant-based foods and reduced consumption of ultra-processed products. Adherence to both Mediterranean and DASH dietary patterns has been associated with a reduced risk of CKD onset, with the DASH diet additionally contributing to reductions in proteinuria and attenuation of the estimated glomerular filtration rate (eGFR) decline [76].

Similarly, data from the human Atherosclerosis Risk in Communities (ARIC) cohort suggest that individuals with the highest adherence to healthy dietary regimens have a 13–20% lower risk of incident CKD than those with the lowest [77].

Targeted nutritional interventions further support the protective role of diet. Low-protein regimens have been shown to reduce proteinuria, while very-low-protein diets are associated with a decreased incidence of end-stage renal disease. Sodium restriction also reduces urinary protein excretion and improves composite renal outcomes.

Plant-based dietary patterns, rich in fibre, phytochemicals, and anti-inflammatory nutrients, appear to confer additional renal benefits, including reductions in proteinuria and improvements in metabolic acidosis, thereby contributing to the preservation of kidney function [77].

Heart-healthy dietary patterns promote both cardiovascular and renal health through shared mechanisms. Greater adherence to DASH, Mediterranean, and plant-based diets is associated with a lower risk of adverse kidney outcomes and reduced cardiovascular events [78].

The convergence of cardiovascular and renal benefits reflects common pathophysiological pathways, including reduced inflammation, improved lipid profiles, better blood pressure control, and decreased insulin resistance [79]. Recent guidelines emphasise that a whole-food, plant-forward dietary approach represents a sustainable and effective strategy for improving both cardiovascular and renal outcomes.

### 6.3. Limitations and Future Directions

Despite growing evidence promoting the role of dietary strategies in influencing inflammation, oxidative stress, and cardiorenal risk in type 2 diabetes mellitus, several limitations should be acknowledged. First, available studies are highly heterogeneous in dietary interventions, comparator groups, follow-up duration, patient phenotypes, baseline metabolic risk, and outcome definitions. This variability limits direct comparison across studies and decreases the strength of pooled evidence.

Second, nutritional assessment is frequently based on self-reported dietary intake, food-frequency questionnaires, or adherence scores, which are intrinsically vulnerable to recall bias, reporting inaccuracies, and interindividual variability in food composition and preparation methods. These limitations are particularly significant when estimating the effects of complex dietary regimens such as the Mediterranean, DASH, or plant-based diets, where the biological effect depends not only on individual nutrients but also on the overall food matrix and long-term adherence.

Third, most interventional studies have relatively short follow-up periods and primarily focus on surrogate outcomes, including glycaemic indices, lipid profiles, inflammatory biomarkers, oxidative stress markers, and anthropometric measures. Although these endpoints are mechanistically informative, they do not necessarily turn into hard cardiovascular or renal outcomes. Long-term trials specifically designed to examine incident cardiovascular events, progression of diabetic kidney disease, heart failure outcomes, and mortality remain limited. A representative synthesis of the main clinical and interventional studies, including their populations, dietary interventions, biomarker changes, clinical outcomes, and key limitations, is provided in Table 3.

Fourth, interpreting inflammatory and oxidative stress biomarkers remains challenging. Many of them, including cytokines, lipid peroxidation products, AGEs, SCFAs, LPS, and TMAO, are affected by analytical variability, sample handling, circadian and postprandial fluctuations, renal function, obesity, smoking, pharmacological therapies, and comorbid inflammatory conditions. Moreover, standardised biomarker panels and clinically validated thresholds remain lacking, limiting their clinical utility.

Fifth, interindividual variability in dietary response remains a major unresolved issue. Genetic background, epigenetic regulation, gut microbiota composition, metabolic phenotype, body composition, renal function, and concomitant glucose-lowering or cardiorenal-protective therapies may all interfere with the biological response to nutritional interventions. Therefore, a uniform dietary prescription may not be enough to evaluate the complexity of type 2 diabetes and its complications.

Future research should move beyond generic comparisons of dietary patterns and prioritise long-term, adequately powered, pragmatic trials that integrate clinical outcomes with mechanistic biomarker assessment. Standardised approaches to dietary assessment, objective measures of adherence, harmonised biomarker panels, and repeated longitudinal sampling are necessary to improve reproducibility and clinical applicability. In addition, multi-omics approaches, including metabolomics, lipidomics, epigenomics, and microbiome profiling, may be useful for identifying diet-responsive molecular signatures and different patients according to their inflammatory, oxidative, metabolic, and cardiorenal risk profiles.

Ultimately, the future direction of nutritional research in type 2 diabetes should focus on developing phenotype-based, biomarker-informed dietary strategies integrated with contemporary pharmacological therapy. Such an approach may help clinicians evaluate which patients are most likely to benefit from specific dietary patterns, which biomarkers are reliable for monitoring response, and how nutritional therapy can be personalised to lower residual cardiovascular and renal risk.

### 6.4. Translational and Clinical Perspectives

From a translational perspective, nutritional therapy should not be considered an alternative to modern glucose-lowering and cardiorenal-protective agents, but rather as the biological and behavioural substrate on which their benefits are well established. Current standards encourage integrating medical nutrition therapy, structured diabetes self-management education and support, physical activity, and psychosocial care with pharmacologic treatment, rather than a sequential model in which lifestyle is considered only before drug escalation. In this framework, GLP-1 receptor agonists and SGLT2 inhibitors should be considered not just as glucose-lowering drugs but also as components of a comprehensive cardio-renal-metabolic strategy whose effectiveness may be amplified or, conversely, limited by dietary quality, body composition, adherence, and baseline inflammatory background [80,81,82].

This integrated view has a direct practical impact on patient selection and counselling. Individuals with obesity, a high appetite drive, or difficulty sustaining caloric restriction may derive particular benefit from combining a Mediterranean- or plant-forward dietary pattern with GLP-1-based therapy, as pharmacologic appetite modulation may increase adherence to higher-quality dietary models. However, these patients should not receive generic dietary advice. Emerging literature suggests that GLP-1-based therapy could be accompanied by reduced dietary variety, gastrointestinal symptoms, micronutrient inadequacy, and loss of lean mass if nutritional follow-up is not structured. In such cases, the nutritional strategy should prioritise protein intake, food quality, symptom-oriented meal adaptation, and preservation of skeletal muscle, particularly in older population, frail individuals, and those with sarcopenic obesity. By contrast, patients with chronic kidney disease, albuminuria, heart failure, or high cardiorenal risk may be especially suitable for SGLT2 inhibitor-based strategies embedded in an overall dietary plan focused on sodium moderation, weight control, and high-quality food patterns, since drug therapy alone does not neutralise the adverse effects of persistent poor diet quality or metabolic inflammation [82,83].

The next translational step is to move from a uniform dietary prescription toward biomarker-informed stratification. Not all individuals with type 2 diabetes have the same pathophysiologic dominance of adiposity, inflammation, ectopic fat accumulation, renal vulnerability, or oxidative stress; therefore, not all are expected to respond similarly to the same dietary modifications. A pragmatic precision nutrition model could begin with routinely available markers such as HbA1c, body weight trajectory, waist circumference, lipid profile, eGFR, and albuminuria, and then progressively consider mechanistic biomarkers such as hs-CRP, IL-6, oxidative stress markers, metabolomic signatures, or gut microbiota-derived metabolites in selected settings. This approach is coherent with the broader precision diabetes medicine agenda, which highlights that treatment selection should increasingly account for heterogeneity in disease mechanisms, prognosis, and treatment response, while remaining clinically feasible and equitable. Notably, recent lipidomic evidence suggests that baseline diet-responsive lipid signatures may identify individuals who derive greater metabolic advantage from improvements in dietary fat quality and from Mediterranean diet interventions, supporting the notion that biomarker-guided nutritional phenotyping is no longer purely theoretical [84,85].

Overall, the clinical challenge is no longer whether nutrition matters in type 2 diabetes, but how to deploy it more intelligently alongside contemporary pharmacotherapy. Future intervention studies should therefore move beyond simple comparisons between diets and instead test integrated care models that consider dietary patterns, drug-class selection, body-composition monitoring, and biomarker trajectories together. Such a strategy would allow clinicians to identify who is most likely to benefit from a GLP-1-centred approach, who should be prioritised for SGLT2 inhibitor-based cardiorenal protection, and who may need intensified nutritional support due to frailty, chronic kidney disease, or a high inflammatory burden. In this sense, precision nutrition should be regarded not as a replacement for guideline-based therapy, but as its natural extension within a truly personalised cardio-renal-metabolic model of care [84,86]. From a practical perspective, nutritional recommendations for patients with type 2 diabetes should prioritise overall dietary quality rather than isolated nutrient restriction. A Mediterranean, DASH-like, or plant-forward dietary pattern should be promoted, with emphasis on vegetables, legumes, whole grains, fruit, nuts, extra-virgin olive oil, fish or other sources of *n*-3 polyunsaturated fatty acids, and adequate dietary fibre intake. Conversely, refined carbohydrates, added sugars, saturated and trans fats, processed meats, and ultra-processed foods should be discouraged due to their associations with postprandial oxidative stress, metabolic inflammation, endothelial dysfunction, and adverse cardiometabolic risk. In patients with chronic kidney disease, nutritional counselling should also include moderation of protein intake, avoidance of excessive sodium intake, and periodic monitoring of renal function and albuminuria. Clinically, dietary intervention should be associated with contemporary glucose-lowering and cardiorenal-protective therapies, particularly GLP-1 receptor agonists and SGLT2 inhibitors when indicated, and should be tailored according to obesity, frailty, sarcopenia risk, renal function, cardiovascular risk, treatment tolerability, and adherence. Routine follow-up should consider body weight and waist circumference, blood pressure, HbA1c, lipid profile, eGFR, albuminuria, and, in selected research or high-risk settings, inflammatory, oxidative, or microbiota-derived biomarkers. This integrated strategy may help turn nutrition-based modulation of inflammation and oxidative stress into clinically meaningful risk reduction.

## 7. Conclusions

Current evidence supports the view that nutrition is not merely an adjunct to glucose control in type 2 diabetes, but a crucial modulator of the inflammatory, oxidative, and metabolic pathways that lead to cardiovascular and renal complications. Across the cardio–renal–metabolic continuum, dietary composition influences endothelial function, mitochondrial homeostasis, gut microbiota composition, and biomarker profiles correlated to disease progression. In this context, Mediterranean, DASH, and plant-based dietary patterns yield the most consistent nutritional models for decreasing low-grade inflammation, oxidative stress, and residual cardio-renal risk. However, the clinical translation of these findings remains not well-defined by heterogeneity in study designs, variable assessments of dietary adherence, short follow-up periods, and incomplete integration of mechanistic biomarkers into intervention studies. Future research should move beyond generic dietary comparisons toward biomarker-informed and phenotype-based therapies that integrate nutrition with contemporary pharmacologic therapies, including GLP-1 receptor agonists and SGLT2 inhibitors. Such an approach may identify a more precise and clinically actionable model of nutrition therapy in diabetes, in which dietary intervention is considered as a core component of personalised cardio–renal risk reduction.

### Use of Generative Artificial Intelligence

In preparing the schematic diagrams for this manuscript, ChatGPT by OpenAI (GPT-5.5 Thinking model, OpenAI, San Francisco, CA, USA; accessed in May 2026)was employed exclusively for AI-assisted graphical refinement and visual organization. The scientific content, conceptual framework, selection of mechanisms, interpretation of pathways, and final structure of the diagrams were developed independently by the authors. All AI-assisted graphical outputs underwent review, editing, and approval by the authors, who assume full responsibility for the accuracy, originality, validity, and integrity of both the figures and the manuscript.

## Figures and Tables

**Table 1:** Key nutrients and dietary patterns modulating inflammatory and oxidative pathways in diabetes, with proposed mechanisms of action.

*Nutrient/Dietary Pattern*	Main Effects	Proposed Mechanisms	References
*Glucose load*	↑ ROS, ↑ inflammatory cytokines	NADPH oxidase activation, NF-κB activation, ↓ IκBα, AP-1/EGR-1 activation	[30,32,33]
*Saturated fats/high-fat meals*	↑ ROS, ↑ endotoxemia, ↑ cytokines	TLR4/CD14 activation, NF-κB signaling, SOCS-3 induction, monocyte activation	[34,35,36]
*Unsaturated fatty acids—n-3 PUFAs and n-6 PUFAs*	↓ inflammation, ↓ liver fat, ↑ insulin sensitivity	Improved lipid metabolism, ↓ PCSK9, and modulation of insulin signalling	[48]
*Dietary fiber*	↓ ROS, ↓ endotoxemia, ↑ metabolic control	SCFA production, FFAR2/3 signalling, ↓ TLR activation, ↑ GLP-1/PYY	[49,50,51]
*Mediterranean diet*	↓ IL-6, TNF-α, oxidative stress; ↑ endothelial function	↓ NF-κB, ↑ NO bioavailability, AMPK/SIRT1 activation, microbiota modulation	[38,39,41]
*DASH diet*	↓ BP, ↓ insulin resistance, ↓ cholesterol	High fibre, low sodium, improved metabolic profile	[42,43]
*Plant-based diet*	↓ CRP, ↓ IL-6	High fibre/phytochemicals, ↓ SFA and AGEs	[44]
*Western diet*	↑ inflammation, ↑ oxidative stress	Refined carbs, SFA, microbiota dysbiosis, endotoxemia	[45]
*Polyphenols (*e.g.*, resveratrol)*	↓ oxidative stress, ↓ inflammation	↑ Nrf2, ↓ NF-κB, ↑ antioxidant enzymes (HO-1, NQO1)	[54]
*Micronutrients (vitamins C, E, Zn, Se)*	↑ antioxidant defense	Support SOD, catalase, GPx, glutathione system	[5,55]

Abbreviations: ↑: increased; ↓: decreased; ROS: reactive oxygen species; NADPH: reduced nicotinamide adenine dinucleotide phosphate; NF-κB: nuclear factor kappa B; IκBα: inhibitor of kappa B alpha; AP-1: activator protein-1; EGR-1: early growth response-1; TLR: toll-like receptor; TLR4: toll-like receptor 4; CD14: cluster of differentiation 14; SOCS-3: suppressor of cytokine signalling-3; *n*-3: omega-3; *n*-6: omega-6; PUFAs: polyunsaturated fatty acids; PCSK9: proprotein convertase subtilisin/kexin type 9; SCFAs: short-chain fatty acids; FFAR: free fatty acid receptor; GLP-1: glucagon-like peptide-1; PYY: peptide YY; IL-6: interleukin-6; TNF-α: tumour necrosis factor alpha; NO: nitric oxide; AMPK: AMP-activated protein kinase; SIRT1: sirtuin 1; BP: blood pressure; CRP: C-reactive protein; SFA: saturated fatty acid; AGEs: advanced glycation end-products; Nrf2: nuclear factor erythroid 2-related factor 2; HO-1: heme oxygenase-1; NQO1: NAD(P)H quinone dehydrogenase 1; Zn: zinc; Se: selenium; SOD: superoxide dismutase; GPx: glutathione peroxidase.

**Table 2:** Established and emerging biomarkers of inflammation and oxidative stress used in nutrition-based intervention studies.

*Biomarker*	Category	Pathophysiological Role	Strengths/ Limitations	References
*IL-6, TNF-α, IL-1β*	Cytokines	Proximal inflammatory signalling, insulin resistance	High biological relevance; high variability, short half-life	[61]
*CRP, SAA*	Acute-phase proteins	Systemic inflammation	Stable, widely used; less mechanistic specificity	[61,62]
*MDA, TBARS*	Lipid peroxidation	Oxidative lipid damage	Easy to measure; limited specificity	[63]
*oxLDL*	Lipoprotein oxidation	Atherogenic oxidative stress	Clinically relevant; influenced by lipid levels	[61]
*F2-isoprostanes*	Lipid peroxidation	Systemic oxidative stress	Gold standard; expensive assays	[61]
*8-OHdG*	DNA oxidation	Oxidative DNA damage	Reflects chronic damage; variability	[61]
*Protein carbonyls*	Protein oxidation	Protein oxidative damage	Stable; influenced by multiple factors	[63]
*SOD, Catalase, GPx*	Antioxidant enzymes	Endogenous defense	Mechanistic insight; sensitive to conditions	[5]
*Paraoxonase-1*	Antioxidant enzyme	Lipoprotein-associated defense	Linked to HDL; variable	[5]
*Myeloperoxidase*	Emerging	Oxidative/inflammatory enzyme activity	Specific, technical variability	[61]
*Nitrotyrosine*	Emerging	Nitrosative stress	Mechanistic relevance; lab complexity	[61]
*4-HNE adducts*	Emerging	Lipid peroxidation signalling	Sensitive marker; assay complexity	[61]
*GSH/GSSG ratio*	Redox balance	Intracellular redox status	Dynamic; sensitive to handling	[61]
*SCFAs*	Microbiota-related	Gut-derived metabolic signalling	Mechanistic; influenced by diet/microbiota	[64]
*Endotoxemia (LPS)*	Microbiota-related	Gut permeability/inflammation	Relevant; assay variability	[65]
*TMAO*	Microbiota-related	Diet–microbiota interaction	Useful in diet comparison; confounders	[64]

Abbreviations: IL: interleukin; IL-6: interleukin-6; IL-1β: interleukin-1 beta; TNF-α: tumour necrosis factor alpha; CRP: C-reactive protein; SAA: serum amyloid A; MDA: malondialdehyde; TBARS: thiobarbituric acid reactive substances; oxLDL: oxidised low-density lipoprotein; F2-isoprostanes: prostaglandin F2-like compounds generated by lipid peroxidation; 8-OHdG: 8-hydroxy-2′-deoxyguanosine; DNA: deoxyribonucleic acid; SOD: superoxide dismutase; GPx: glutathione peroxidase; HDL: high-density lipoprotein; GSH: reduced glutathione; GSSG: oxidised glutathione; GSH/GSSG: reduced-to-oxidised glutathione ratio; SCFAs: short-chain fatty acids; LPS: lipopolysaccharide; TMAO: trimethylamine N-oxide; 4-HNE: 4-hydroxynonenal.

**Table 3:** Selected Clinical and Interventional Studies Evaluating Nutrition-Based Strategies in Diabetes and Related Cardiorenal Outcomes.

Study	Population	Intervention	Duration	Biomarkers Changed	Clinical Outcomes + Main Limitation
[75]	7447 adults at high cardiovascular risk; many had type 2 diabetes or multiple cardiometabolic risk factors.	Mediterranean diet + extra-virgin olive oil or nuts versus control advice on fat reduction.	Median 4.8 years	Not a biomarker-driven primary report; ancillary analyses suggest a more favourable oxidative/inflammatory profile.	Reduced major cardiovascular events versus control. Limitation: primary-prevention cohort not restricted to diabetes; biomarker assessment not standardised in the main report.
[39]	1002 patients with established coronary heart disease, including a relevant subgroup with abnormal glucose metabolism/type 2 diabetes.	Mediterranean diet versus low-fat diet in secondary prevention.	7 years	Inflammatory/oxidative biomarkers were not systematically reported as main endpoints in the event paper.	The Mediterranean diet was superior to a low-fat diet in preventing major cardiovascular events. Limitation: single-centre secondary-prevention trial.
[41]	Patients with type 2 diabetes mellitus.	Mediterranean diet-based interventions versus control dietary approaches.	Varied across included studies.	mprovements in glycaemic control, body weight, and cardiovascular risk factors.	Supports the beneficial role of Mediterranean diet-based interventions in type 2 diabetes. Limitation: heterogeneity of included interventions and reliance on surrogate metabolic outcomes rather than hard cardiovascular or renal endpoints.
[43]	Adults with type 2 diabetes mellitus.	DASH diet versus ADA nutrition-guideline-based diabetic diet.	12 weeks	Triglycerides, total cholesterol, VLDL, and free fatty acids decreased in both groups; DASH adherence was associated with favourable short-term cardiometabolic changes.	Supports the short-term metabolic benefit of DASH-style counselling in type 2 diabetes. Limitation: short intervention and no cardiovascular/renal hard endpoints.
[70]	Meta-analysis of 7 interventional studies including 338 participants with type 2 diabetes.	Intermittent fasting versus standard dietary approaches.	Short-term; varied across studies	Greater weight loss with intermittent fasting; no additional HbA1c reduction versus standard diets.	Intermittent fasting may improve weight in the short term, but evidence for superior glycemic benefit remains weak. Limitations: small sample sizes and heterogeneous protocols.
[65]	Meta-analysis of 22 randomised controlled trials in adults with chronic disease/risk states.	Mediterranean, DASH, and vegetarian/vegan dietary patterns versus control diets.	Varied across trials	The Mediterranean diet showed the most consistent reductions in IL-6, IL-1β, and CRP; effects were less robust for DASH and vegetarian/vegan patterns.	Strongly supports the biomarker dimension of dietary modulation of inflammation. Limitation: heterogeneous populations/interventions and no hard endpoints.
[59]	Patients with diabetes and chronic kidney disease.	Moderate dietary protein intake, approximately 0.8 g/kg/day, versus excessive protein intake	Not applicable	Not primarily biomarker-based; aimed at reducing renal haemodynamic stress and slowing CKD progression	Supports moderate protein intake as part of diabetic kidney disease management. Limitation: recommendation-based entry rather than a dedicated interventional trial
[77]	Prospective cohort of >14,000 middle-aged adults evaluating plant-based diet scores and kidney outcomes.	Healthy plant-based patterns versus less healthy plant-based patterns.	Long-term follow-up	No repeated inflammatory biomarker panel was central to the analysis; healthier plant-based patterns tracked with slower eGFR decline.	Higher adherence to healthy plant-based diets was associated with favourable kidney outcomes and a lower risk of incident CKD. Limitation: observational design and population not restricted to diabetes.

Abbreviations: ADA, American Diabetes Association; CKD, chronic kidney disease; CRP, C-reactive protein; DASH, Dietary Approaches to Stop Hypertension; eGFR, estimated glomerular filtration rate; GFR, glomerular filtration rate; HbA1c, glycated haemoglobin; HDL, high-density lipoprotein; IL, interleukin; VLDL, very-low-density lipoprotein.

## Data Availability

Data are contained within the article.
